# AUP1 Regulates the Endoplasmic Reticulum-Associated Degradation and Polyubiquitination of NKCC2

**DOI:** 10.3390/cells13050389

**Published:** 2024-02-24

**Authors:** Nadia Frachon, Sylvie Demaretz, Elie Seaayfan, Lydia Chelbi, Dalal Bakhos-Douaihy, Kamel Laghmani

**Affiliations:** 1 Centre de Recherche des Cordeliers, INSERM, Sorbonne Université, Université de Paris, F-75006 Paris, France; nadia.frachon@sorbonne-universite.fr (N.F.); sylvie.demaretz@sorbonne-universite.fr (S.D.); elie.seaayfan@uni-marburg.de (E.S.); dalal.douaihy@gmail.com (D.B.-D.); 2 CNRS, ERL8228, F-75006 Paris, France

**Keywords:** kidney, ion transport, ER quality control, ERAD, AUP1, Bartter syndrome, CKD

## Abstract

Inactivating mutations of kidney Na-K-2Cl cotransporter NKCC2 lead to antenatal Bartter syndrome (BS) type 1, a life-threatening salt-losing tubulopathy. We previously reported that this serious inherited renal disease is linked to the endoplasmic reticulum-associated degradation (ERAD) pathway. The purpose of this work is to characterize further the ERAD machinery of NKCC2. Here, we report the identification of ancient ubiquitous protein 1 (AUP1) as a novel interactor of NKCC2 ER-resident form in renal cells. AUP1 is also an interactor of the ER lectin OS9, a key player in the ERAD of NKCC2. Similar to OS9, AUP1 co-expression decreased the amount of total NKCC2 protein by enhancing the ER retention and associated protein degradation of the cotransporter. Blocking the ERAD pathway with the proteasome inhibitor MG132 or the α-mannosidase inhibitor kifunensine fully abolished the AUP1 effect on NKCC2. Importantly, AUP1 knock-down or inhibition by overexpressing its dominant negative form strikingly decreased NKCC2 polyubiquitination and increased the protein level of the cotransporter. Interestingly, AUP1 co-expression produced a more profound impact on NKCC2 folding mutants. Moreover, AUP1 also interacted with the related kidney cotransporter NCC and downregulated its expression, strongly indicating that AUP1 is a common regulator of sodium-dependent chloride cotransporters. In conclusion, our data reveal the presence of an AUP1-mediated pathway enhancing the polyubiquitination and ERAD of NKCC2. The characterization and selective regulation of specific ERAD constituents of NKCC2 and its pathogenic mutants could open new avenues in the therapeutic strategies for type 1 BS treatment.

## 1. Introduction

Cell injury secondary to prolonged ER stress is considered a major contributor to the pathophysiology of several serious human diseases, including chronic kidney disease (CKD) [1,2]. Kidneys have to adapt to a wide range of cellular stressors, such as hypoxia, oxidative stress, and proteostasis disturbances [1,2]. These diverse cellular stressors may lead to dysfunction of the ER, which is required to preserve protein homeostasis by keeping the balance between protein-folding capacity and protein-folding load [1]. In renal cells, including glomerular and epithelial tubular cells, an imbalance in ER proteostasis leads to the generation and progression of kidney disorders [1,3]. Given that the ERAD system is dedicated to the clearance of unwanted misfolded proteins from the ER, this cellular machinery plays a pivotal role in maintaining ER proteostasis [3,4]. Consequently, extensive studies of the molecular pathways underlying the ERAD system have provided several new insights into how ERAD is a major contributor to human pathologies [5,6]. Accordingly, the ERAD pathway was reported to be involved in a wide range of pathologies called conformational diseases, including CKD, such as diabetic insipidus and Bartter’s syndromes [1,7,8,9]. For instance, it was reported that mutations in ROMK1 that are related to type 2 BS lead to the ER retention and ERAD of this potassium channel [8]. Likewise, we previously demonstrated that type 1 BS is also related to the ERAD system [9]. Type 1 BS is an inherited serious kidney disease, diagnosed essentially during the prenatal stage due to the detection of polyhydramnios, polyuria, salt loss, and electrolyte abnormalities [10,11]. Type 1 BS patients may not survive the early prenatal stage without the appropriate treatment [12]. Type 1 BS results from inactivating mutations in NKCC2 [10,13]. Similar to ROMK1 mutants [8], we recently showed that all studied NKCC2 mutations enhanced the ERAD of the cotransporter hampering therefore NKCC2 cell surface expression and function [9]. In addition, we previously provided evidence that mutations in *MAGED2* also enhance the EARD of NKCC2 and the related renal cotransporter NCC, consequently causing a very severe type of antenatal BS characterized by massive polyhydramnios, perinatal death, and extreme prematurity [14,15]. Together, all these data highlighted the fact that NKCC2 transit through the ER represents a pivotal step in the forward trafficking of the cotransporter to the cell surface. Hence, our findings emphasized the need to further characterize the ERAD constituents of NKCC2 to potentially identify new treatment strategies for type 1 BS. 

The ERAD system involved in the removal of unwanted proteins depends essentially on the site of protein misfolding detected by molecular chaperones, which takes place either in the cytoplasm, the ER membrane, or the ER lumen [5,16,17]. Similar to other members of cation-Cl cotransporters, NKCC2 and NCC are composed of twelve membrane-spanning domains and two large cytoplasmic regions [11,18]. Hence, these transmembrane proteins can interact with the ER molecular chaperones on either edge of the ER membrane. Also, given that the amino-terminal and carboxyl-terminal tails of NKCC2 are the major cytoplasmic regions of the cotransporter, they are most likely the main sites of protein-protein interaction (PPIs). In support of this notion, several PPIs involving NKCC2 N-terminus were reported [19,20]. Likewise, we previously identified several NKCC2 protein partners interacting with NKCC2 C-terminus and modulating the cotransporter intracellular trafficking [21,22,23,24,25]. For instance, we reported that OS9, STCH, and Hsp70 bind to NKCC2 immature form at the ER to regulate its ERAD [23,24]. In this study, we report a novel PPI between the NKCC2 COOH terminus and AUP1. Interestingly, AUP1 is also an interactor of the protein-lectin OS9 [26], a key member of the ERAD components of NKCC2 [23]. Here, we show that, similar to OS9, AUP1 binds to the ER-resident form of NKCC2 and enhances its proteasomal degradation. Moreover, we provide evidence that AUP1 promotes the polyubiquitination of the cotransporter, revealing, therefore, a new mode of NKCC2 molecular regulation. Consequently, these findings provide novel insights into the ER quality control (QC) of NKCC2 proteins and, therefore, could pave the way to the discovery of new treatment strategies for renal disorders caused by defects in the expression and trafficking of the cotransporter. 

## 2. Materials and Methods

### 2.1. Yeast Two-Hybrid Assay

Yeast two-hybrid (Y2H) screening was carried out as described in our previous studies [21,23]. In the present study, we used the cDNA segment encoding the intermediate domain of NKCC2 carboxy-terminal tail (residues 741–909) as bait. Briefly, the yeast strain AH109 expressing this bait was mated with the Y187 yeast strain pre-transformed with a human kidney cDNA library. The selection of positive clones was carried out by growing mated yeast cells on high stringency selection mediums (−Leu, −Trp, −His, −Ade) followed by a β-galactosidase activity test [21].

### 2.2. Plasmid Constructions and Site-Directed Mutagenesis 

Generation Myc-NKCC2, Myc-NCC, and eGFP-NKCC2 constructs were previously described [21,23]. The human AUP1 cDNA was subcloned into pcDNA3.1/V5 and pcDNA3.1⁄CT-GFP expression vectors (Invitrogen, Paris, France) to produce AUP1-V5 and AUP1-GFP constructs. To generate the nontagged version of AUP1, a codon stop was inserted at the end of the AUP1 encoding sequence using WT AUP1-V5 construct by site-directed mutagenesis according to the Quik Change protocol (Stratagene, Les Ulis, France). The HA-tagged ubiquitin plasmid (PMT123) was generously provided by Dr. Dirk Bohmann (University *of* Rochester).

### 2.3. Cell Culture

Opossum kidney cells (OKP cells) and human embryonic kidney (HEK) cells were maintained in DMEM (Gibco 42430, Paris, France) complemented with 10% fetal bovine serum (Eurobio, Les Ulis, France), streptomycin, and penicillin at 37 °C with 5% CO_2_ [23,24,25]. Transient transfection was conducted as previously described [20,21,24] using Lipofectamine plus kit (Invitrogen, Paris, France). MG132 and kifunensine (Sigma, Saint-Quentin-Fallavier, France) were used at 7.5 μM and 25 µM, respectively, for 6 h before cell lysis.

### 2.4. Immunoprecipitation, Immunocytochemistry and Biotinylation

Immunoprecipitation (IP) and immunocytochemistry experiments were carried out as described in detail in our previous studies [22,23,24,25]. Briefly, IP was performed using the primary antibody of interest, and affinity purification was performed using protein G-agarose beads (Dynabeads from Invitrogen, Paris, France). For immunocytochemistry studies, cells were fixed with 2% paraformaldehyde at room temperature. After cell permeabilization with 0.1% Triton X-100, DAKO was used for 30 min to block nonspecific binding before incubation with the primary antibodies for 1 h at room temperature. The primary antibodies used are as follows: mouse anti-Myc (Takara, Clontech, Saint-Germain-en-Laye, France) mouse anti-V5 (Invitrogen, Paris, France), rabbit anti-Calnexin (Abcam, Paris, France), and rabbit anti-AUP1 (Sigma, Saint-Quentin-Fallavier, France). The *Zeiss* LSM 710 (Carl Zeiss Co. Ltd., Seoul, Republic of Korea) laser scanning confocal microscope was used to analyze the stained specimens. For cell surface expression assessment, we conducted a cell surface biotinylation assay in detail, as reported earlier [21,22,23]. Briefly, cells were incubated at 4 °C for 1 h with NHS-biotin (1 mg/mL). Total cell extracts were then incubated with NeutrAvidin-agarose beads (Thermo Fisher, Montigny le Bretonneux, France ) overnight at 4 °C before centrifugation at 16,000 rpm. The resulting pellets, representing biotinylated plasma membrane proteins, were used for Western blotting (WB). Proteins were detected using an ECL detection kit (PerkinElmer Life Sciences, Villebon-sur-Yvette, France). 

### 2.5. Cycloheximide-Chase Assays

The maturation and degradation of NKCC2 were assessed by cycloheximide (CHX) chase assay, as reported previously [23,27]. CHX (100 μM) was added to the culture medium 14–16 h after transfection with NKCC2. Cell lysates were collected at 0, 1, 2, and 4 h after CHX treatment and used for WB.

### 2.6. Small Interfering RNA (siRNA)

AUP1 and OS9 siRNAs were bought from Dharmacon (L-012410-01-0005 and L-010811-01-0005, respectively). Before NKCC2 transient transfection, HEK cells were first transfected with siRNAs using Lipofectamine RNAiMAX (Invitrogen, Paris, France) [23,24,25]. Then, 24–48 h later, cell lysates were collected to perform WB analysis. 

### 2.7. Statistical Analyses

Results are presented as mean ± S.E. To compare the differences between means, *t*-tests or ANOVA were used appropriately. With *p* < 0.05, the difference was judged statistically significant. 

## 3. Results 

### 3.1. Identification of AUP1 as a Novel NKCC2-Interacting Protein

**Figure 1:**
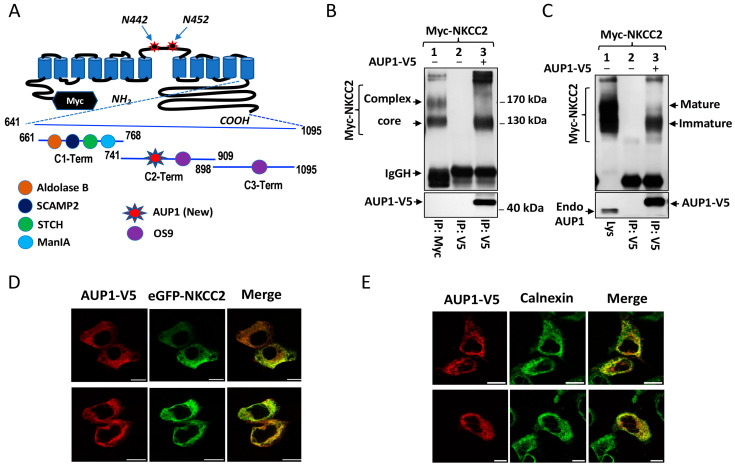
Identification of AUP1 as a novel binding partner of NKCC2. (**A**) Y2H baits. A proposed model of Myc-NKCC2 topology is shown. As reported previously [21], we used three regions of NKCC2 COOH terminus as baits for our Y2H screening. Aldolase B, SCAMP2, STCH, and ManIA bind to C1-term [21,22,24,25], whereas OS9 interacts with C3-term [23]. We report here the interaction of OS9 and AUP1 with C2-term. (**B**,**C**) AUP1 binds to NKCC2 in OKP cells (**B**) and HEK cells (**C**). Lysates from cells transfected with Myc-NKCC2 alone or with AUP1-V5 were used for immunoprecipitation (IP) with anti-Myc (lane 1, **B**) or anti-V5 antibodies (lanes 2 and 3). *Lys*, total cell lysate. NKCC2 and AUP1 were revealed by IB using Myc (**B**,**C**), V5 (**B**), and AUP1 antibodies (**C**), respectively. IgGH, the heavy chain of IgG. (**D**,**E**) AUP1 co-localizes with NKCC2 at the ER. (**D**) After transfection with eGFP-NKCC2 and AUP1-V5, cells were immunostained with mouse anti-V5 (conjugated with Texas Red-coupled secondary antibody). The merge color (yellow) illustrates an overlap between the eGFP-NKCC2 protein (green) and AUP1-V5 (red). (**E**) OKP cells were immunostained with mouse anti-V5 (Texas red) and rabbit anti-calnexin (FITC, green). The merge color illustrates the colocalization of the proteins. Bars, 10 μm.

To uncover novel interactors of NKCC2, we previously used the yeast two-hybrid (Y2H) system to screen a kidney cDNA expression library, using three baits (C1-term, C2-term and C3-term) covering the predicted NKCC2 COOH terminus [21] (Figure 1A). In contrast to C1-term [21,22,24,25] and C3-term [23], the identity of the proteins interacting with C2-term (residues 741–909) remains to be revealed. Among the positive clones of our Y2H screening using C2-term as bait, one matched the sequence of AUP1 (clone #27) (Figure 1A). Interestingly, in addition to AUP1, two positive clones (clones #34 and #48) encoding for OS9 were also isolated (Figure 1A). This is of great interest since not only did we previously identify OS9 as a binding partner of NKCC2-C3-term [23], but also because OS9 was reported to be an interactor of AUP1 [26]. We demonstrated earlier that OS9 interacts with full-length NKCC2 in vivo [23]. Thus, as with OS9, we tested whether NKCC2 protein might also bind to AUP1 in a cellular environment. Toward that end, OKP cells were transfected with Myc-NKCC2 with or without AUP1-V5. Cell lysates were used for immunoprecipitation (IP) using anti-V5 antibody, and the resultant immunoprecipitates were analyzed by immunoblotting (IB). As illustrated in Figure 1B, lane 3, the cotransporter protein was easily recovered from AUP1 immunoprecipitates, witnessing robust interaction of the proteins. Interestingly, similar to OS9 [23], AUP1 binding to NKCC2 involves primarily the high-mannose (immature) form of the cotransporter. Given that PPI may depend on the cell type used, we next tested AUP1 interaction with NKCC2 in HEK cells. Importantly, AUP1 interacts again mainly with NKCC2 immature form in HEK cells (Figure 1C). It is noteworthy that, without co-transfection of NKCC2 with AUP1-V5, the cotransporter protein was not detected in anti-V5 immunoprecipitates (Figure 1B,C, lane 2), strongly indicating that the interaction of the proteins is specific. Taken together, these data provide evidence that AUP1 binds to NKCC2, an association that engages only the cotransporter immature form.

The association of AUP1 only with the ER-resident form of NKCC2 indicates that this interaction occurs at the ER. To verify this, we evaluated the subcellular distribution of eGFP-NKCC2 and AUP1-V5 by immunofluorescence confocal microscopy. It is worth noting that we previously documented that tagging NKCC2 N-terminus with eGFP does not affect NKCC2 forward trafficking to the plasma membrane [21,23]. As can be seen in Figure 1D, eGFP-NKCC2 was nicely colocalized with AUP1-V5. Interestingly, similar to OS9 [23], the immunofluorescence staining pattern of the two colocalized proteins was largely confined to a perinuclear ER-like distribution. Moreover, AUP1 was extensively colocalized with calnexin (Figure 1E), demonstrating thereby that the site of NKCC2 binding to AUP1 is the ER. Of note, NKCC2 interaction with AUP1 at the ER is consistent with the fact that AUP1 also interacts with the ER lectin OS9 [26].

### 3.2. Comparison of the Effect of AUP1 with That of OS9 on NKCC2

**Figure 2:**
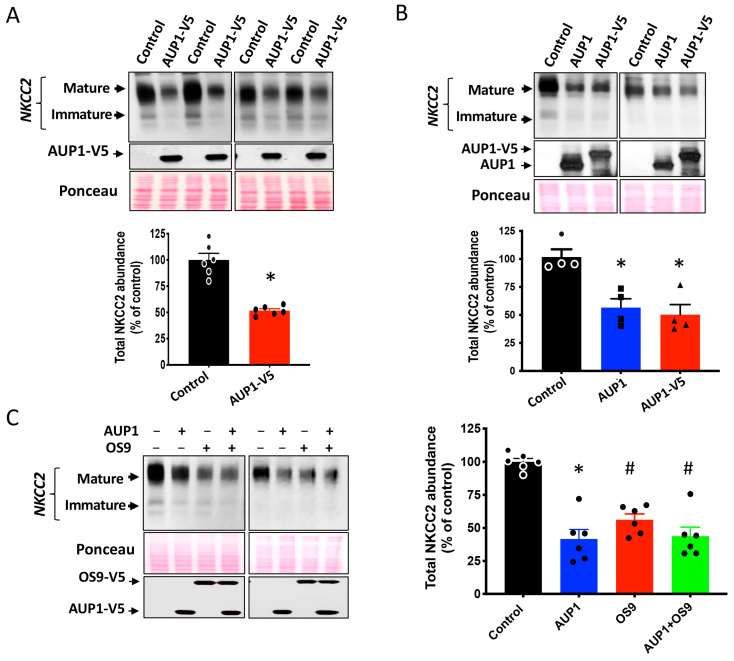
AUP1 and OS9 similarly regulate NKCC2 protein expression. (**A**) Overexpressing AUP1 decreases NKCC2 protein abundance. (**Upper panel**), representative Western blot illustrating AUP1 effect on NKCC2 in OKP cells. NKCC2 was transfected in cells alone (0.2 μg/well) or with AUP1-V5 (0.6 μg/well); 48 h post-transfection, Western blotting was carried out using Myc-NKCC2 and AUP1-V5. (**Lower panel**), quantitative analysis of the cotransporter amounts. Results were normalized with the loading control (Ponceau staining). * *p* < 0.0001 (n = 6) versus control. (**B**) Untagged AUP1 regulates also NKCC2. (**Upper panel**), effect of untagged and V5-tagged AUP1 on NKCC2. Cells were cotransfected with Myc-NKCC2 alone (control) or with AUP1 or AUP1-V5. (**Lower panel**), summary of results. * *p* < 0.006 (n = 4) versus control. (**C**) AUP1 and OS9 effects on NKCC2. (**Left panel**), effect of AUP1 and/or OS9 co-expression on NKCC2. Cells were cotransfected with NKCC2 (0.2 μg/well) alone or with AUP1-V5 and/or OS9-V5 (0.6 μg/well). (**Right panel**)*:* summary of data. *, *p* < 0.0001; #, *p* < 0.0002; versus control (n = 6).

Previous studies documented that AUP1 is an important component of the ERAD machinery [26,28,29,30,31]. Moreover, given that OS9 also interacts with NKCC2 immature form at the ER and enhances its ERAD [23], we anticipated that AUP1 also plays a key role in the ER QC of the cotransporter. Hence, to elucidate the functional relevance of AUP1-NKCC2 interaction, we first sought to check the effect of AUP1 overexpression on the amount of NKCC2 protein. As shown in Figure 2A, in OKP cells overexpressing AUP1-V5, NKCC2 protein abundance was strikingly decreased. Importantly, the same effect was reproduced when NKCC2 was cotransfected with untagged AUP1 protein (Figure 2B), clearly indicating that the AUP1-induced decrease in NKCC2 protein level is not related to protein overexpression of the V5-tagged version of AUP1. We then compared the AUP1 effect to that of OS9 on NKCC2. To that end, cells were transfected with NCCC2 alone or with AUP1-V5 and/or OS9-V5. Interestingly, OS9 and AUP1 had a similar effect on NKCC2 (Figure 2C)**,** strongly suggesting that similar to OS9 [23], AUP1 also promotes the ERAD of the cotransporter. Notably, in cells simultaneously transfected with both NKCC2 partners (AUP1 + OS9, Figure 2C), the effects of AUP1 and OS9 were not additive, implying that both proteins act in the same pathway to regulate NKCC2 biogenesis. Moreover, it is of great interest to mention that, in contrast to the observed decrease in NKCC2 protein expression, AUP1 overexpression did not affect OS9 protein abundance (Figure 2C), strongly suggesting that AUP1 action on the cotransporter is specific. It is noteworthy that overexpressing aldolase B or SCAMP2, two other interactors of NKCC2 C-terminus, did not affect total NKCC2 protein abundance, [21,22], further confirming, therefore, the specificity of NKCC2 regulation by AUP1. 

### 3.3. AUP1 Is a Common Regulator of NKCC2 and NCC 

**Figure 3:**
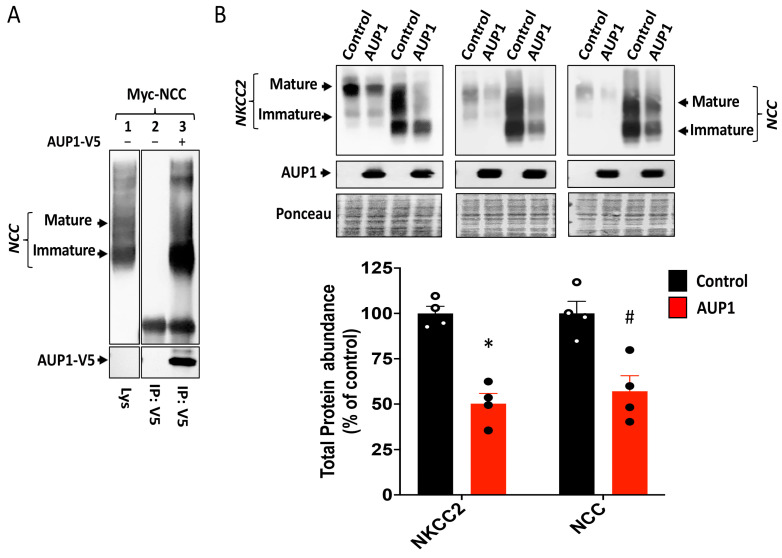
AUP1 is a common regulator of NKCC2 and NCC. (**A**), interaction of AUP1 with NCC in OKP cells. Cells were transfected with Myc-NCC alone or with AUP1-V5. Co-immunoprecipitated NCC with AUP1-V5 was revealed by IB using anti-Myc (lane 3)*. Lys*, total cell lysate. (**B**), AUP1 regulates similarly NKCC2 and NCC. (**Upper panel**), effect of AUP1 on NKCC2 and NCC. OKP cells were transfected with NKCC2 or NCC (0.2 μg/well) with or without AUP-V5 (0.6 μg/well). (**Lower panel**), quantitative analysis of NKCC2 and NCC protein abundance. *, *p* < 0.0007; #, *p* < 0.007; versus control (n = 4).

Given that the carboxyl-terminus of NKCC2 has a high degree of homology with the C-terminal region of other members of the sodium-dependent chloride cotransporter family [11], we postulated that AUP1 may be a common protein partner and regulator of all members of this family. To verify this hypothesis, we checked whether AUP1 interacts with the related kidney-specific Na-Cl cotransporter NCC. To this end, Myc-NCC was transiently expressed in OKP cells singly or with AUP1-V5. Of note, like NKCC2, we previously showed that when Myc-NCC is transiently transfected into OKP cells, both the high mannose (immature) and fully glycosylated (mature) forms of the cotransporter are detected [23]. Interestingly, similar to NKCC2, NCC was also detected in AUP1 immunoprecipitates (Figure 3A, lane 3). Moreover, similar to NKCC2, AUP1 binds mainly to NCC immature form (Figure 3A, lane 3). Hence, we next studied the relevance of AUP1 interaction with NCC by comparing the effect of AUP1 on NCC protein amount with that on NKCC2. Similar to NKCC2, NCC protein expression was also decreased in OKP cells overexpressing AUP1 (Figure 3B). Hence, these data strongly indicate that AUP1 is a common regulator of Na-dependent chloride cotransporters. 

### 3.4. AUP1 Decreases NKCC2 Maturation and Cell Surface Expression

**Figure 4:**
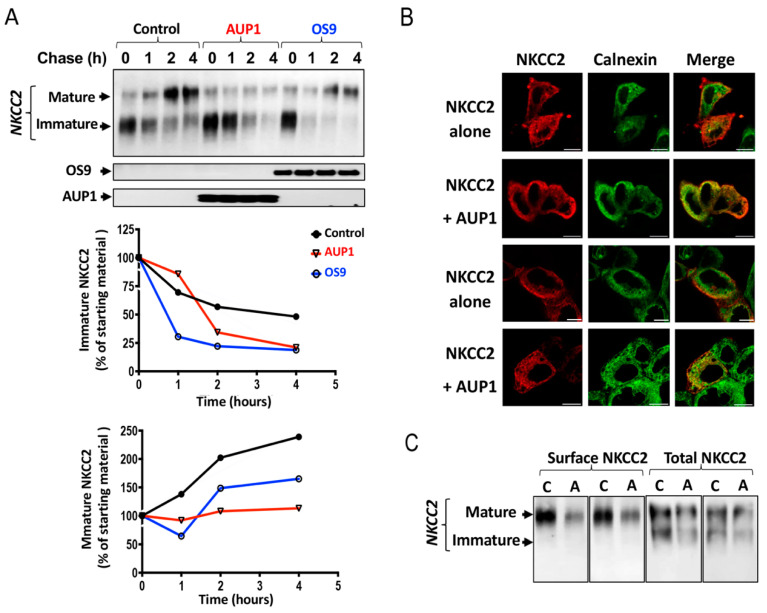
AUP1 impairs NKCC2 maturation and trafficking to the plasma membrane: (**A**) Monitoring of the maturation and degradation of NKCC2 using CHX-chase assay following AUP1 or OS9 overexpression. (**Upper panel**), IB illustrating AUP1 and OS9 effects on NKCC2 stability and maturation. 14–16 h after cell transfection, total cell lysates were collected at the indicated time points after CHX treatment (100 μM) to perform IB with Myc and V5 antibodies. (**Lower panel)**, quantitative analysis of NKCC2 bands. (**B**) Effect of AUP1 on NKCC2 subcellular distribution. Myc-NKCC2 and the ER marker calnexin were detected by mouse anti-Myc (Texas Red; red) and rabbit anti-calnexin (FITC; green). The merge color (yellow) illustrates the colocalization of the cotransporter with the ER marker. Bars, 10 μm. (**C**) Effect of AUP1 on surface and total NKCC2. OKP cells were transfected with NKCC2 alone (**C**) or with AUP1 (**A**). Total and biotinylated (surface) NKCC2 were detected by IB using anti-Myc.

Similar to OS9, given that AUP1 interacts only with the ER-resident form of NKCC2, the reduction in the cotransporter protein level most likely results from alterations in NKCC2 maturation and/or stability. To test this hypothesis, we compared the effect of AUP1 to that of OS9 on NKCC2 maturation and degradation using CHX assay as described previously [23,27]. As expected, when NKCC2 was transfected alone, its core glycosylated form was converted to the fully glycosylated form during the chase period (Figure 4A). In agreement with our previous reports [23], OS9 co-expression massively increased the degradation of NKCC2 decreasing, therefore, its maturation (Figure 4A). Most importantly, AUP1 co-expression also enhanced the degradation of immature NKCC2 and severely hampered the maturation of the cotransporter (Figure 4A, upper and lower panels). Together, these results clearly show that AUP1 binds to NKCC2’s immature form to promote its efficient ERAD, heavily impairing thereby the cotransporter maturation.

To elucidate the consequence of AUP1-induced downregulation of NKCC2, we checked its impact on NKCC2 subcellular distribution. As expected, when expressed alone, NKCC2 protein was found to be distributed primarily to the cell plasma membrane, and its immunostaining surrounded the ER marker (calnexin) signal (Figure 4B). In contrast, in cells overexpressing AUP1, a major portion of NKCC2 proteins was trapped in the ER as illustrated by extensive colocalization of the cotransporter with calnexin (ER marker) (Figure 4B). As a consequence, the amount of NKCC2 at the cell surface was greatly diminished under these conditions. To further confirm the effect of AUP1 on cell surface NKCC2, we used the cell surface biotinylation method [21,22,23]. Of note, we previously documented that only the fully glycosylated (mature) form of NKCC2 is capable of reaching the cell plasma membrane [21,23]. Fittingly, only mature NKCC2 was detected in biotinylated plasma membrane proteins (Figure 4C). Most importantly, the AUP1-induced decrease in the amount of total NKCC2 was associated with a massive reduction in NKCC2 expression at the plasma membrane (Figure 4C). Together, these data provide evidence that AUP1 promotes efficient ERAD of NKCC2, hindering thereby NKCC2 maturation and forward trafficking to the cell surface.

### 3.5. AUP1 Enhances the ERAD of NKCC2 in a Proteasome-Dependent Fashion

**Figure 5:**
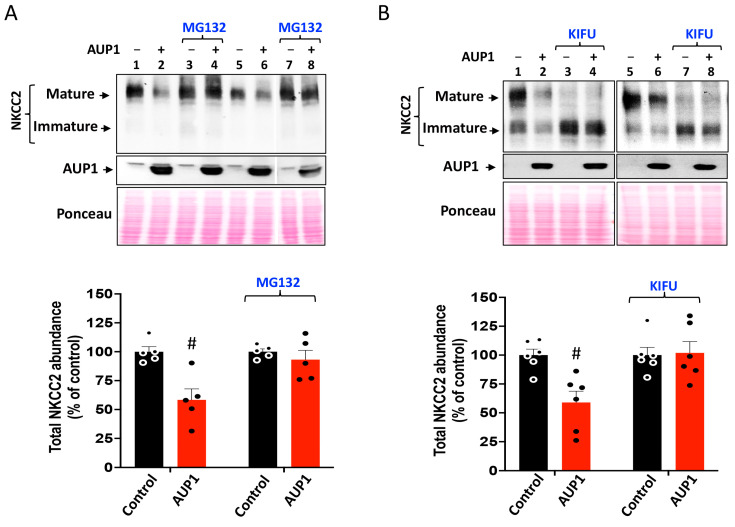
AUP1 enhances NKCC2 ERAD in a proteasome-dependent manner. (**A**) AUP1 effect on NKCC2 involves the proteasome pathway; 16 h post-transfection, OKP cells were treated with MG132 for 6 h before cell lysis. (**Upper panel**), total cell lysates were used for Western blotting to reveal NKCC2 and AUP1 proteins. (**Lower panel**), quantitative analysis of the cotransporter bands. #, *p* < 0.05 versus control (n = 5). (**B**) Mannose trimming is necessary for the AUP1 effect on NKCC2; 16 h post-transfection, cells were treated with kifunensine (KIFU) for 6 h before cell lysis. (**Upper panel**), Detection of NKCC2 and AUP1 proteins by WB using Myc and V5 antibodies. (**Lower panel**), densitometric analysis of NKCC2 bands. #, *p* < 0.05 versus control (n = 6).

Given that the OS9 effect on the ERAD of NKCC2 involves mainly proteasome pathways [23], we next explored the implication of this degradation pathway in AUP1-induced downregulation of NKCC2. To achieve this, 16 h post-transfection, cells were incubated with the proteasome inhibitor MG132 for 6 hours. As expected, MG132 massively augmented the amount of immature NKCC2 without affecting the cotransporter maturation (Figure 5A) [23]. Most importantly, in cells overexpressing AUP1, MG132 fully abolished AUP1-induced NKCC2 degradation (Figure 5A). Taken together, these results indicate that AUP1 promotes efficient degradation of the cotransporter in a proteasome-dependent fashion.

Given that N-glycans are crucial in the ERAD of glycoproteins [24,32,33], we also examined kifunensine action on AUP1-induced reduction in the cotransporter protein expression. Kifunensine is commonly used as an inhibitor of the ERAD pathway by inhibiting ER and Golgi α-mannosidases [24,32,33,34,35]. Expectedly, kifunensine disrupted NKCC2 complex glycosylation (Figure 5B). Most importantly, kifunensine fully abolished AUP1-induced downregulation of NKCC2, clearly indicating that mannose trimming is required for AUP1 effect on the cotransporter (Figure 5B). Hence, these data prove that AUP1-enhanced degradation of NKCC2 is N-glycan dependent and further confirm the presence of NKCC2 ERAD pathway mediated by AUP1.

### 3.6. Regulation of NKCC2 by Endogenous AUP1

**Figure 6:**
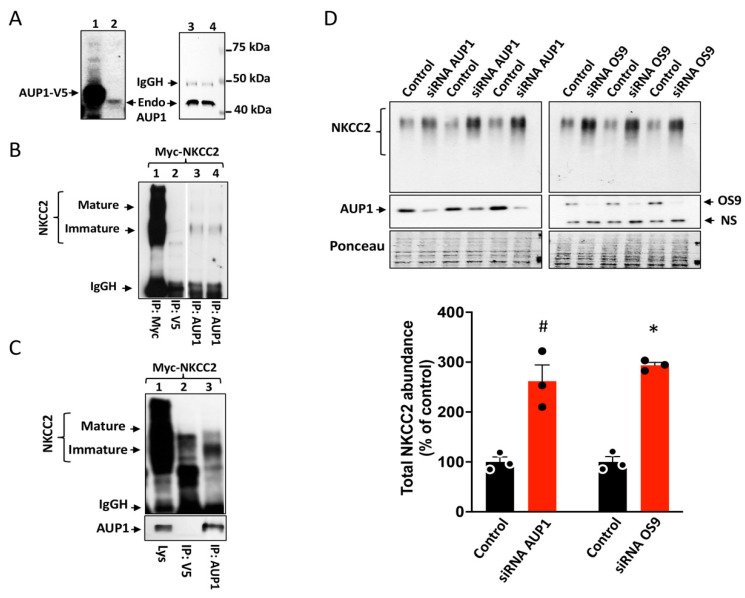
Endogenous AUP1 regulates NKCC2 protein expression. (**A**) endogenous expression of AUP1 in OKP cells. Lysates from OKP cells transfected with AUP1-V5 (lane 1) or non-transfected cells (*lane 2*) were used for IB analysis using the AUP1 antibody. Total cell extracts from OKP cells were subjected to *IP* using anti-AUP1, and the resultant immunoprecipitates were analyzed by IB using the same antibody (*lanes 3 and 4*). (**B**) endogenous AUP1 associates with immature NKCC2. Protein immunoprecipitation was carried out using anti-Myc (positive control, lane 1), anti-V5 (negative control; lane 2), or anti-AUP1 (lanes 3 and 4). Co-immunoprecipitated immature NKCC2 was detected by Myc antibody (lanes 3 and 4). (**C**) endogenous AUP1 binds also to immature NKCC2 in HEK cells. *Lys*, total cell lysate. (**D**) The knockdown (KD) of OS9 and AUP1 similarly increased NKCC2 protein abundance. (**Upper panel**), IB illustrating the effect of OS9 and AUP1 KD on total NKCC2. HEK cells stably expressing NKCC2 were transfected with specific siRNAs for AUP1 or OS9 or nonspecific siRNAs (Control); 48 h post-transfection, total cell extracts were used for IB to detect NKCC2, AUP1, and OS9 proteins. NS, nonspecific. (**Lower panel**), summary of data. #, *p* < 0.001; *, *p* < 0.05, (n = 3).

Using mainly protein overexpression studies, we generated consistent data pointing toward the presence of an ERAD pathway of NKCC2 mediated by AUP1. However, one can always argue that the observed effects are due, at least in part, to protein overexpression. Consequently, we sought to study the role of endogenous AUP1 in the regulation of NKCC2 protein expression. First, to discount the possibility that the interaction described above (Figure 1B,C) is secondary to nonspecific binding because of protein overexpression, we next checked whether endogenous AUP1 also interacts with NKCC2. For this purpose, we used an antibody produced against native AUP1 protein. To test the efficiency of this antibody in detecting AUP1 in OKP cells, we subjected cell lysates obtained from non-transfected OKP cells and cells transfected with AUP1-V5 to Western blotting using this anti-AUP1 antibody. In cells overexpressing AUP1-V5, a band of approximately 45 kDa, corresponding to the predicted size of V5 tagged AUP1 protein, was revealed, clearly witnessing the efficiency of this antibody to recognize AUP1 (Figure 6A (lane 1)). Importantly, a single band around 40 kDa, consistent with the expected size of AUP1 protein, was also obtained from non-transfected cells (Figure 6A, lane 2). Moreover, a better signal of that band around 40 kDa was obtained after IP using anti-AUP1 (Figure 6A, lanes 3 and 4). Collectively, these data provide evidence that AUP1 is endogenously expressed in OKP cells. More importantly, in OKP cells overexpressing NKCC2, only the immature form of the cotransporter was co-immunoprecipitated with endogenous AUP1, indicating specific physical interactions of the proteins (Figure 6B, lanes 3 and 4). In addition, NKCC2 was not recovered from anti-V5 immunoprecipitates (Figure 6B, lane 2), further confirming the specificity of the interaction (Figure 6B, lane 2). Finally, similar to OKP cells, endogenous AUP1 also binds to NKCC2 in HEK cells, an interaction that involves, again, only the cotransporter ER-resident form (Figure 6C, lane 3).

Second, to rule out the possibility that the effect of AUP1 on NKCC2 is simply an artifact of protein overexpression, we took advantage of the availability of our HEK cell line stably expressing NKCC2 [36] to corroborate our findings by studying the role of endogenous AUP1 in the regulation of the cotransporter. Toward that, we tested, in parallel, the impact of AUP1 and OS9 knockdown on the amount of NKCC2 protein. To this end, HEK cells, stably expressing NKCC2, were transfected with siRNAs of AUP1 or OS9 for at least 24 hours before cell lysis. In agreement with our previous report, OS9 knockdown increased the protein level of NKCC2 immature and mature forms (Figure 6D), leading, therefore, to an increase in total cellular NKCC2 protein [23]. Most importantly, similar to OS9, AUP1 knockdown enhanced NKCC2 protein expression, indicating, therefore, that endogenous AUP1 also regulates NKCC2 biogenesis. Together with the data generated by protein overexpression, these findings further corroborate our conclusion that AUP1 is an enhancer of NKCC2 ER-associated degradation. 

### 3.7. Effect of AUP1 on NKCC2 Polyubiquitination

**Figure 7:**
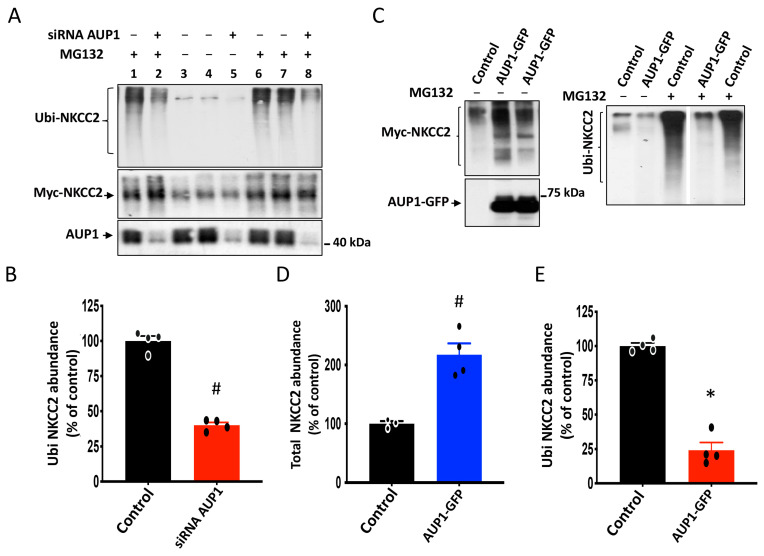
Inhibition of endogenous AUP1 increases NKCC2 polyubiquitination. (**A**) AUP1 knockdown decreases NKCC2 polyubiquitination. HEK cells transiently cotransfected with Myc-NKCC2 and HA-Ub plasmids, with or without AUP1 siRNAs, were treated with or without MG132. Anti-Myc antibody was used to immunoprecipitate NKCC2, and Western blots were probed with HA antibody to reveal ubiquitinated NKCC2 (Ubi NKCC2). An aliquot of total cell lysate was used to detect Myc-NKCC2 and endogenous AUP1 proteins using Myc and AUP1 antibodies. (**B**) quantitative analysis of polyubiquitinated NKCC2 proteins from cells overexpressing NKCC2 alone or with AUP1 siRNA. #, *p* < 0.004 (n = 4). (**C**) Effect of AUP1 dominant negative on NKCC2. **Upper panel**, effect of AUP1-GFP overexpression on total and polyubiquitinated NKCC2. OKP cells transiently cotransfected with Myc-NKCC2 and HA-Ub plasmids, with or without AUP1-GFP, were treated with or without MG132. After Myc-NKCC2 immunoprecipitation, samples were probed to detect Ubi NKCC2 using anti-HA antibodies. An aliquot of total cell lysate was used to detect Myc-NKCC2 and AUP1-GFP proteins using Myc and AUP1 antibodies. (**D**,**E**), summary of data. *, *p* < 0.0001 versus control (n = 3). #, *p* < 0.004, versus control (n = 4).

Polyubiquitination of ERAD substrates is a key step in proteasomal degradation, and previous reports have shown that AUP1 is involved in this process [26,31]. Hence, we sought to check the effect of AUP1 on the cotransporter polyubiquitination. First, to check whether NKCC2 is regulated by polyubiquitination, Myc-NKCC2 and HA-Ub plasmids were cotransfected in HEK293 cells and were treated without or with the proteasome inhibitor MG132. Anti-Myc antibody was used to immunoprecipitate NKCC2, and Western blots were probed with anti-HA antibody to reveal polyubiquitinated NKCC2 (Ubi NKCC2). As can be seen in Figure 7A (lanes 3, 4, and 5), in the absence of MG132, polyubiquitinated proteins were barely detected. By contrast, the presence of MG132 dramatically increased NKCC2 polyubiquitination, as illustrated by the presence of a smear appearing in the range from 130 to 250 KDa (Figure 7A, lanes 1, 2, 6–8). Hence, these data indicate that polyubiquitination followed by proteasomal degradation contributes to the regulation of NKCC2. Most importantly, the amount of polyubiquitinated NKCC2 was strikingly decreased following AUP1 knockdown (Figure 7A, lanes 1, 2, 6–8, and Figure 7B), strongly suggesting that AUP1 promotes the ERAD of NKCC2 by enhancing its polyubiquitination.

In addition to siRNA interference assay, previous reports indicated that studying the role of endogenous AUP1 can be performed using C-terminally tagged AUP1 with GFP, considered as a dominant negative mutant of the protein [26,37]. Consequently, we sought to further confirm our findings that endogenous AUP1 regulates NKCC2 biogenesis by assessing the effect of AUP1-GFP on total and polyubiquitinated NKCC2 protein levels in OKP cells. As can be seen in Figure 7C,D, in cells overexpressing AUP1-GFP, NKCC2 protein abundance was increased, which is in line with the effect of AUP1 knockdown on the cotransporter. Hence, these data are consistent with the idea that AUP1-GFP behaves as a dominant negative variant of AUP1 and strongly hint, again, at a role of endogenous AUP1 in the ERAD of immature NKCC2. Moreover, similar to AUP1 knockdown in HEK cells, cotransfecting NKCC2 with AUP1-GFP in OKP cells resulted in a marked decrease in NKCC2 polyubiquitination (around 85%) (Figure 7C,E) clearly indicating that AUP1 enhances the ubiquitin-dependent proteasome of NKCC2, independently of the expression system. 

### 3.8. AUP1 Promotes Efficient Degradation of NKCC2 Folding Mutants

**Figure 8:**
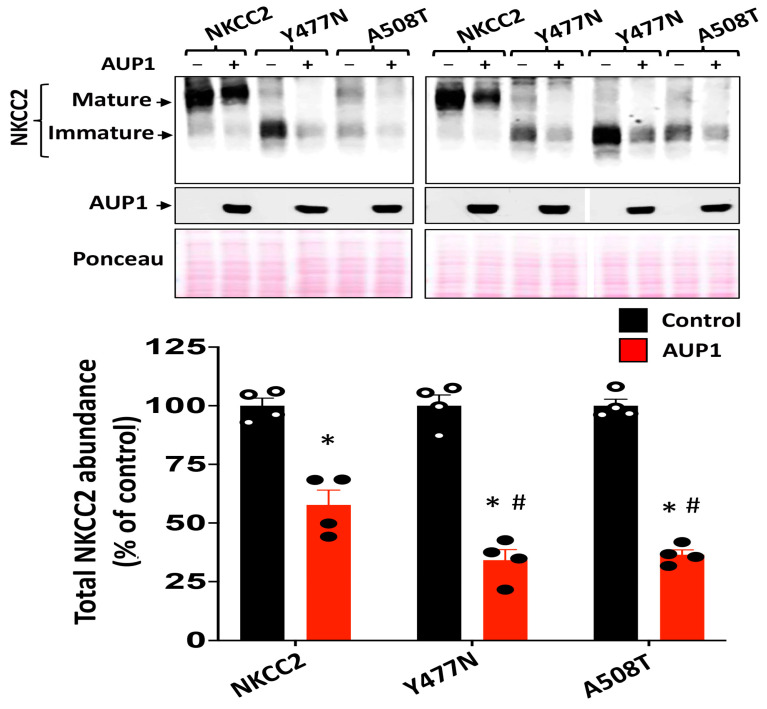
AUP1 promotes the efficient degradation of NKCC2 disease-causing mutants. **Upper panel**, IB illustrating AUP1 effect on NKCC2 mutants Y477N and A508T. OKP cells were transfected with NKCC2, Y477N, or A508T, with or without AUP1. AUP1 and NKCC2 were detected by Western blotting using Myc and V5 antibodies **Lower panel**, densitometric analysis of NKCC2 bands. *, *p* < 0.005, (n = 4) versus control. #, *p* < 0.05 (n = 4) versus (WT NKCC2 + AUP1) group.

We previously reported that type 1 BS is related to the ERAD system [9]. Hence, to further corroborate our conclusion that AUP1 is a key player in the ERAD of NKCC2, we next checked AUP1’s impact on Y477N and A508T, two type 1 BS mutants [9,38]. Of note, we demonstrated previously that Y477N and A508T are trapped at the ER [9]. To check whether, similar to WT NKCC2, Y477N and A508T are regulated by AUP1, we compared the effects of AUP1 on these NKCC2 mutants with that on WT NKCC2. As illustrated in Figure 8, similar to WT NKCC2, overexpressing AUP1 decreased Y477N and A508T protein levels. Most importantly, AUP1 co-expression had a more pronounced effect on A508T and A477N, strongly indicating that AUP1 promotes the efficient degradation and clearance of NKCC2 disease-causing mutants.

## 4. Discussion

The main purpose of the present work was to provide novel insights into the molecular pathways governing the regulation of NKCC2 ERAD. Using the Y2H system and co-immunoprecipitation assays, we identified AUP1 as a novel interactor of the immature and ER-resident form of NKCC2. We have uncovered that AUP1 knockdown enhances the amount of NKCC2 protein while its overexpression produces the opposite result by decreasing the cotransporter stability and maturation. Importantly, we have also found that AUP1 promotes the polyubiquitination of NKCC2, which is a key step in the ERAD system. Interestingly, our data also revealed that NKCC2 disease-causing mutants are more prone to the AUP1-mediated ERAD pathway. These new findings could help to better understand how defects in the cotransporter expression and trafficking cause type 1 BS, which is crucial to elucidate the pathophysiology of this salt-losing tubulopathy and upgrade the available treatments.

Early investigations identified AUP1 as a cytosolic protein with a role in integrin signaling [26,39]. However, more detailed immunofluorescence analysis revealed that AUP1 was localized to the ER membrane [37] as well as to lipid droplets [40]. Moreover, two previous studies reported that overexpression of AUP1 dominant negative impaired the degradation of MHC class I heavy chain and tail-anchored UBC6e [37,41], which is consistent with an active functional role for AUP1 in the ERAD system. In support of this idea, it has been reported that AUP1 interacts at the ER with the Hrd1-SEL1L ubiquitin ligase complex [26,37] and the ERAD-related E2 Ube2g2 [31,37]. Interestingly, the AUP1 protein adopts a hairpin-like topology in the ER membrane with both the N- and C-terminal of the protein facing the cytosol [42]. Consequently, AUP1 interaction with the ERAD component machinery occurs mainly at the cytoplasmic side of the ER. In this regard, Klemm, et al., reported that AUP1 also binds to the ER lectin OS9 [26], a key member of the NKCC2 ERAD system. It is worthy of note that previous reports have suggested that in mammalian cells, OS9 might be located at both luminal and cytoplasmic edges of the ER [43,44,45]. Hence, an interaction between AUP1, OS9, and the cotransporter at the cytoplasmic surface of the ER is possible. Consistent with this, we demonstrated that, similar to OS9 [23], AUP1 is involved in the NKCC2 ERAD machinery. Of note, we have already identified several ERAD components of NKCC2, such as OS9 and STCH [23,24]. However, the identification of an interactor of NKCC2 directly involved in the polyubiquitination step of the cotransporter ERAD was never reported. As with OS9 and STCH, AUP1’s implication in the ERAD of the cotransporter was initially hinted at by Co-IP experiments revealing that the association of the proteins engages primarily the ER-resident form of NKCC2. In support of a direct role of AUP1 in the proteasome-dependent ERAD pathway of NKCC2, we demonstrated that in cells overexpressing AUP1, the downregulation of NKCC2 protein level was fully prevented in the presence of the proteasome inhibitor MG132 and the mannose trimming inhibitor kifunensine. Moreover, we also provided evidence that in the presence of MG132, the increase in the cotransporter protein level following inhibition of endogenous AUP1 was associated with a marked decrease in polyubiquitinated NKCC2 proteins, which is consistent with the notion that AUP1 enhances the ubiquitin-dependent proteasome degradation of NKCC2.

The accumulation of misfolded protein aggregates in the ER is harmful to cellular health [6,46,47]. To preserve protein homeostasis, in particular, in the secretory pathway, eukaryote cells rely on several distinct quality control (QC) checkpoints between the ER and the plasma membrane [5,48,49,50]. Thus, misfolded proteins, especially transmembrane proteins such as NKCC2 and NCC, are thoroughly examined by consecutive QC check spots, such as the ER QC and Golgi QC, before reaching the cell surface [49,51]. Fittingly, we previously reported that the vast majority of newly synthesized NKCC2 proteins are destined for ERAD via the proteasomal and lysosomal pathways and that this process is more accentuated with NKCC2 mutants [14,23,24]. Moreover, we reported previously that in addition to ER quality control involving OS9 and STCH, a Golgi QC involving Golgi Mannosidase IA (ManIA) at the cis Golgi compartment may contribute to the ERAD of the cotransporter by trapping misfolded proteins that escaped ER QC and bringing them back to the ER [25]. Importantly, we demonstrated here that AUP1 also has a more profound impact on the fate of NKCC2 folding mutants when compared to WT NKCC2, strongly suggesting that AUP1 is a new key player in the ERAD of NKCC2 mutants in type 1 BS. Our data strongly suggest, therefore, that AUP1 works in concert, sequentially or simultaneously with OS9, STCH, and ManIA by promoting the polyubiquitination of the cotransporter, to contribute to the adequate clearance of harmful misfolded NKCC2 proteins, safeguarding thereby cells from proteotoxicity generated by the accumulation of misfolded proteins, especially during the disease state. 

Identification of the protein partners of NKCC2 ER-resident form is crucial for the characterization of the cotransporter ERAD machinery. Most importantly, the characterization of the ERAD constituents of NKCC2 could help in the rescue of its ER-retained mutants by preventing their interactions with the ER QC components of the cotransporter. In this regard, we recently showed that the processing of NKCC2 disease-causing mutants can be rescued, at least partially, by chemical chaperones such as glycerol and the ER stress reliever 4-PBA [9]. However, the effect of glycerol and 4-PBA on the expression of the mature and functional form of NKCC2 was small, and therefore, we were not able to fully rescue the transport activity of NKCC2 [9]. Likewise, OS9 knockdown protected the immature form of mutants NKCC2 from ERAD but failed to rescue the mature and functional form of the cotransporter [9]. These data further corroborate the notion that more intensive research is required to fully understand the maturation and function of NKCC2 mutants, probably by using combinations of chemical chaperones and/or molecular chaperones such as AUP1, STCH, OS9, and Golgi ManIA. The notion of combining the effect of multiple compounds has proven to be one of the best strategies to fully rescue the processing and function of the CFTR mutant ΔF5408 [52,53,54]. We are currently exploring these possibilities. 

Even though our work was conducted in renal cultured cells, it is tempting to postulate that NKCC2 and NCC associations with AUP1 and OS9 could also occur in native kidney cells and contribute to the chronic regulation of the cotransporters in vivo. Consistent with this, a previous report showed that knocking out Nedd4-2, a protein partner of NCC, strikingly increased the cotransporter protein level, thereby causing salt-sensitive hypertension in animals [55,56]. Likewise, Trudu, et al., demonstrated that patients with an abnormally increased level of uromodulin, a binding partner of NKCC2, develop a furosemide-sensitive salt-dependent hypertension [36]. Hence, one can also anticipate that changes in AUP1 and OS9 abundance could also modify the expression of the co-transporters in vivo. In line with this hypothesis, previous studies have reported that AUP1 and OS9 are up-regulated in response to ER stress and facilitate the ERAD of glycoproteins in a cellular context of ER stress [57,58]. In this regard, the AUP1 and OS9 overexpression approach used in our study may mimic, therefore, AUP1 and OS9 upregulation under situations generating ER stress in renal cells, such as chronic high salt intake and aging [59,60]. This is of particular interest since NKCC2 protein expression is decreased in the aging kidney and during high salt loading [61,62]. Hence, upregulation of AUP1 and/or OS9 could contribute to the reduction in the cotransporter expression via the ubiquitin-proteasome pathway in an ER stress environment [61,62]. 

In summary, we found that NKCC2 binds to AUP1, a protein known to be involved in the ERAD system. AUP1 interacts mainly with the ER-resident form of NKCC2 to accelerate its ERAD by enhancing its polyubiquitination. To the best of our knowledge, this is the first report describing the polyubiquitination of the cotransporter as a new mode of NKCC2 molecular regulation. Besides NKCC2, AUP1 also binds to the structurally related cotransporter NCC and regulates its protein expression, suggesting that the interaction with AUP1 is a common feature of members of cation-chloride cotransporters, a group of proteins that are targets of therapeutic drugs and mutated in several human pathologies such as Gitelman, Bartter, and Andermann syndromes [11,63,64]. The in-depth characterization of the molecular pathways underlying the ERAD of the SLC12A family members may provide a foundation for the discovery of novel therapeutic strategies targeting their transport from the ER to the cell surface.

## Data Availability

The data that support the findings of this study are available on request from the corresponding author.
